# A comprehensive analysis of the uterine microbiome in endometrial cancer patients - identification of *Anaerococcus* as a potential biomarker and carcinogenic cofactor

**DOI:** 10.3389/fcimb.2025.1511625

**Published:** 2025-01-29

**Authors:** Olga Kuźmycz, Aleksandra Kowalczyk, Aleksandra Bolanowska, Anna Drozdzowska, Jakub Lach, Wiktoria Wierzbińska, Tomasz Kluz, Paweł Stączek

**Affiliations:** ^1^ Department of Molecular Microbiology, Institute of Microbiology, Biotechnology and Immunology, University of Lodz, Faculty of Biology and Environmental, Protection, Lodz, Poland; ^2^ Department of Gynecology and Obstetrics, Fryderyk Chopin University Hospital No. 1, Rzeszow, Poland; ^3^ Biobank Lab, Department of Cancer Biology and Epigenetics, University of Lodz, Faculty of Biology and Environmental Protection, Lodz, Poland; ^4^ BioMedChem Doctoral School of the University of Lodz and Lodz Institutes of the Polish Academy of Sciences, Lodz, Poland; ^5^ Department of Gynecology, Gynecology Oncology and Obstetrics, Institute of Medical Sciences, Medical College of Rzeszow University, Rzeszow, Poland

**Keywords:** endometrial cancer, microbiome, *Anaerococcus vaginalis*, 16S rRNA metagenomics, ANCOM analysis

## Abstract

**Introduction:**

Endometrial cancer (EC) is a significant gynecological malignancy with increasing incidence worldwide. Emerging evidence highlights the role of the uterine microbiome in the pathogenesis of EC. This study aims to characterize the uterine microbiome in EC patients and identify potential microbial biomarkers, with a focus on *Anaerococcus* as a differentiating taxon.

**Methods:**

The endocervical canal swabs from patients with EC (n=16) and non-cancerous patients (EM, n=13) were collected. The V3-V4 region of the 16S rRNA gene was sequenced using the Illumina platform. Bioinformatic analyses were performed with QIIME2, and statistical comparisons were conducted to assess differences in microbial composition and diversity. *In vitro* experiments were conducted to assess the functional impact of *Anaerococcus* on human uterine fibroblasts, including its ability to adhere to the human cells and induce oxidative stress.

**Results:**

The α-diversity metrics, including Shannon entropy and observed amplicon sequence variants (ASVs), revealed significantly higher microbial diversity in EC samples compared to EM. *Anaerococcus* was identified as a key taxon differentiating EC from EM groups, showing a higher relative abundance in EC samples. Functional predictions and *in vitro* assays indicated that *Anaerococcus* may contribute to carcinogenesis by inducing reactive oxygen species (ROS) production, and has the high ability to adhere to the human endometrial fibroblasts.

**Discussion:**

The study provides evidence of distinct microbial signatures in EC, with *Anaerococcus* emerging as a potential biomarker. The *in vitro* findings suggest its role in endometrial carcinogenesis, underscoring its potential as a target for future diagnostic and therapeutic applications.

## 1 Introduction

Endometrial cancer (EC) is one of the most common female reproductive tract malignancies in developed countries. According to the International Agency for Research on Cancer, the incidence rate of EC is increasing rapidly compared with 2018 and is estimated to increase by more than 50% worldwide by 2040. In 2020, Poland had the highest rate of EC in the world, which corresponded to 9,869 diagnosed new cases (Chen et al., 2017; Morice et al., 2016; World Cancer Research Fund International, 2023). Only several factors, including host genetic alterations and hereditary factors, have been shown to play important roles in endometrial carcinogenesis. But still, this can only explain 10–20% of EC cases. A woman’s lifetime risk of EC is approximately 3%, with a median age at diagnosis of 61 years. Environmental factors, such as hormones, obesity, inflammation, as well as menopausal status and microbiome composition, were found to be related to EC initiation and progression (Kuźmycz and Stączek, 2020; Morice et al., 2016). The Human Microbiome Project has revealed that about 9% of the total human microbiome was found in the female reproductive tract. Historically, the cervix was considered to be the barrier which protected the upper genital tract from bacteria. Thus, the uterus, in its physiological state, was suggested to be a bacteria-free zone. However, the studies with the use of metagenome sequencing techniques demonstrated a diversity of bacterial populations in the uterus, which additionally can undergo significant changes in pathological states (Moreno et al., 2022). Interestingly, a risk factor for EC is postmenopause since, in this period, an increase of uterine bacterial diversity is observed, which is associated with disorders and pathologies in the female reproductive tract. The endometrial microbiota in postmenopausal women may create conditions that allow the bacterial community correlated to EC to emerge. It was suggested that EC-related bacteria are probably associated with chronic inflammation and disruption of host cellular functions, leading to a carcinogenic process (Medina-Bastidas et al., 2022; Walsh et al., 2019). In the presented study, the endocervical canal microbiomes of women with EC or endometrial myoma (EM) were examined to reveal the differences in microbial composition. Some pathological taxa were identified, which may play a crucial role in EC development and progression. Previous studies have linked species such as *Prevotella* and *Gardnerella* to uterine dysbiosis in endometrial cancer (EC). However, their association with other gynecological and systemic diseases, such as bacterial vaginosis (BV) and pelvic inflammatory disease (PID) (Gilbert et al., 2019; Qing et al., 2024), challenges their utility as specific diagnostic markers for EC. In contrast, the role of *Anaerococcus* in the uterine microenvironment and EC pathogenesis remains unexplored. This study provides a preliminary evaluation of *A. vaginalis*’s capacity to induce reactive oxygen species (ROS) production and adhere to endometrial cells—two critical processes that may underlie its involvement in cancer progression. By focusing on these initial interactions, we aim to position *Anaerococcus* as a potential microbiological biomarker specific to EC, distinguishing it from previously studied genera. Importantly, our work represents a novel perspective on this microorganism and highlights its potential diagnostic relevance.

## 2 Materials and methods

### 2.1 Participant enrollment

In this study, we analyzed the endocervical canal microbiomes of the 29 participants. The endocervical canal swabs were prepared and supplied by the Fryderyk Chopin Clinic no 1 of Rzeszow, Poland. The procedure of material identification and collection was supported with all necessary protocols and procedures and obtained the Regional Bioethics Committee approval number 24/B/2019 with further updates. Participants were patients of the Gynecology Department of Fryderyk Chopin Clinical Hospital no 1 in Rzeszow who were qualified for surgical treatment due to diagnosed EC or EM. The mainstay of treatment for EC is total hysterectomy with bilateral salpingo-oophorectomy. Hysterectomy and adnexectomy can be done with minimally invasive techniques (laparoscopy or robotassisted surgery), vaginally, or laparotomically (Braun et al., 2016; Morice et al., 2016). Removal of the uterus is also recommended for women with symptomatic EM, for whom medical treatments have failed, and who have completed childbearing (Thubert et al., 2016). The main criteria for the selection of participants were as follows: women aged 18 years or older, with EC or EM, who had not been treated with antibacterial agents for more than one month before collecting the samples. Participants also completed the additional questionnaires, the data of which are presented in **Table 1**. Continuous variables were compared using the Mann-Whitney U test, and categorical variables were analyzed using Fisher’s exact test. A p-value < 0.05 was considered statistically significant.

**Table 1:** Patient demographics.

Variables	Endometrial myoma (n= 13)	Endometrial cancer (n=16)	P-value
**Age (years) - median, IQR**	48.6 (42-53)	63.6 (51-78)	**0,002**
**Caucasian ethnicity (%)**	13 (100)	16 (100)	–
**Age of the first menstruation (years) – median, IQR**	13,8 (10-17)	13,8 (11-17)	–
**Postmenopausal status**	100%	100%	–
**Number of births – median, IQR**	2.1 (0-4)	2.1 (0-5)	0,765
**Miscarriages**	2	0	0,474
**Antibiotics for last month**	0	0	0,043
**Antibiotics for last year**	2	2	1,000
**HRT** **Yes** **No**	0 13	1 15	1,000
**PCOS** **Yes** **No**	0 13	1 15	1,000
**Endometriosis** **Yes** **No**	1 12	1 15	1,000
**Wight – median, IQR**	72.44 (55-82)	80.92 (57-115)	0,098
**BMI – median, IQR**	26 (20-31)	30 (24-38)	0,051
**Diabetes** **Yes** **No**	1 12	4 12	0,324
**Smoking status** **Yes** **No**	1 12	1 15	1,000
**Thyroid abnormalities** **Yes** **No**	0 13	5 11	**0,043**
**Breast cancer** **Yes** **No**	0 13	1 13	1,000

IQR – interquartile range; HRT, hormone replacement therapy; PCOS, polycystic ovary syndrome; BMI, body mass index.

Statistical analyses were performed using Mann-Whitney U for nonparametric data categorical variables were analyzed using Fisher’s exact test. The significant differences are marked in bold (p < 0.05).”

The study was approved by the Bioethics Committee of the Regional Medical Council in Rzeszow City (Protocol number 24/B/2019, update 54/B/2020, date of approval 21.05.2020).

### 2.2 Endocervical canal swabs collection

Swabs were collected by the surgeon from the endocervical canal (after visualization in a speculum) before the hysterectomy using sterile Dacron swabs and then placed in sterile Falcon tubes. The smear material prepared in this way was stored on dry ice. For long-term storage, -80°C was used. No additional buffer was added to the swabs.

### 2.3 Sample preparation and processing

**Figure 1:**
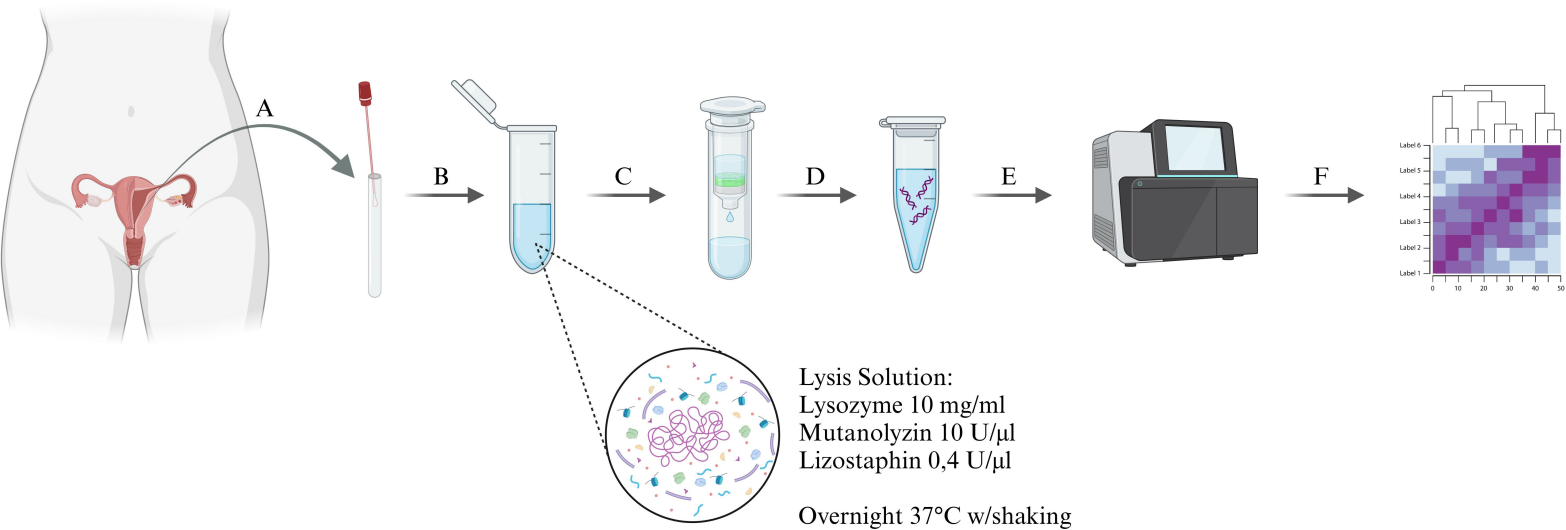
Diagram of the experimental procedure, **(A)** – sample collection, **(B)** – primary lysis in Lysis Solution, containing the specific bacterial enzymes cocktail, **(C)** - bacterial DNA extraction, **(D)** – bacterial DNA amplification, **(E)** – sequencing using the Illumina MiSeq next-generation sequencing platform, **(F)** – bioinformatic analysis of the samples.

1 ml of the sterile Dulbecco′s Phosphate Buffered Saline (DPBS) was added to the swabs and then vortexed three times for 15 seconds. Samples were then centrifuged at 10,000 x g for 15 minutes to collect all bacterial cells in the supernatant. All genomic DNA extractions were performed using the DNeasy Power Soil Kit (Qiagen, Hilden, Germany) with minor modifications. For lysis, Lysis Solution was used, which was a part of the kit supplemented with Lysozyme (10 mg/ml), Mutanolyzin (10 U/µl), and Lizostaphin (0.4 U/µl) (AandA Biotechnology, Gdańsk, Poland). Lysis was performed overnight at 37°C with gentle shaking. Subsequent DNA extraction steps were performed according to the manufacturer’s instructions. The amount of eluted DNA was measured on BioDrop (BioDrop Ltd, Harvard Bioscience, Hollistone, USA. MA). The 5 ng of extracted DNA was used for polymerase chain reaction (PCR) amplification of the V3-V4 region of 16S rDNA. The reaction was prepared according to the instructions for 16S Metagenomic Sequencing Library Preparation (Illumina, San Diego, USA. CA) and the Nextera XT DNA Library Preparation Kit (Illumina, San Diego, USA. CA). 16S rDNA sequencing was performed by the University of Lodz Biobank Lab using the Illumina MiSeq next generation sequencing platform (Illumina, San Diego, USA. CA) (**Figure 1**).

### 2.4 Bioinformatic sequence analysis

In this study, 16S metagenomics targeting the V3-V4 region was employed rather than full metagenomic sequencing. This approach was chosen for its high resolution in analyzing microbial communities and cost-effectiveness for exploratory studies. At the first stage of analysis, the quality of reads was checked using FastQC. In the next step, adaptors and low-quality sequences were removed from the reads with trim galore v. 0.6.4 set on default parameters. Further analysis was performed with QIIME2 2021.4. DADA2 was used for denoising data and ASVs (amplicon sequence variants) table generation with parameters –p-trim-left-f 25 –p-trunclen-f 240 –p-trim-left-r 25 –p-trunc-len-r 240. Alpha and beta diversity metrics were generated with the core-metrics-phylogenetic plugin with a sampling depth of 27 132. Alpha diversity, which presents the evenness and richness of bacterial population within samples, was investigated with the Shannon index, Pielou’s evenness and ASVs number. Between groups, a comparison of α-diversity was performed with the Kruskal-Wallis test. Beta diversity was measured by three methods: Bray-Curtis dissimilarity, Unweighted UniFrac distance and Weighted UniFrac distance. These indexes were calculated for analysis of the shared diversity between bacterial communities in terms of ecological distance between samples. Differences in β-diversity between groups were tested by the PERMANOVA procedure, which is a multivariate analysis of variance based on distance matrices and permutation. Principal coordinate analysis (PCoA) of β-diversity was also performed and plotted with QIIME2. The Analysis of Compositions of Microbiomes (ANCOM) was performed using the standard QIIME2 pipeline.

The purpose of alpha and beta diversity analyses was to assess variability and composition of the microbiome in EC and EM patient samples. The Shannon index was used to evaluate the evenness and richness of bacterial populations, while beta diversity was analyzed using Unweighted and Weighted UniFrac metrics and Bray-Curtis distances. The results indicate significant differences in microbiota composition between groups, potentially reflecting the pathological role of the microbiome in EC.

### 2.5 Human uterine fibroblasts cell culture

Human uterine fibroblasts (HUF; C12385) were purchased from PromoCell and cultured in Fibroblast Growth Medium 2 (23020 PromoCell, Heidelberg, Germany) supplemented with 2% fetal bovine serum (FBS, PromoCell, Heidelberg, Germany) and 1x streptomycin/penicillin solution (Cytogen, Zgierz, Poland). The culture was performed at 37°C, 5% CO_2_. Prior to the experiment, subculture was performed in Basal Medium Eagle (BME, Cytogen, Zgierz, Poland) supplemented with 2% FBS without antibiotics. Subculture was performed under normoglycemic conditions by adding 5 mM D-(+)-glucose solution (Merck, Darmstadt, Germany) to the culture medium.

### 2.6 Bacterial culture and MOI determination

*Anaerococcus vaginalis* (DSM no. 7457, Leibniz Institute DSMZ-German Collection of Microorganisms and Cell Cultures GmbH, Braunschweig, Germany) was anaerobically cultured in tryptic soy broth (TSB; BioMaxima S.A., Lublin, Poland) complemented with 5% defibrinated horse blood (BioMaxima S.A., Lublin, Poland), vitamin K1 (ThermoFisher Scientific, Waltham, USA. MA), and HAEMIN (ThermoFisher Scientific, Waltham, USA. MA)), solutions. *Lactobacillus jensenii* (DSM no. 20557, Leibniz Institute DSMZ-German Collection of Microorganisms and Cell Cultures GmbH, Braunschweig, Germany), used as a physiological control, was cultured in TSB containing 10% FBS at 37°C and 5% CO_2_. The multiplicity of infection (MOI) was determined by direct infection of human uterine fibroblasts (10,000 cells/well) with bacteria from each double-dilution. Subsequently, the viability of human uterine cells was analyzed 24 hours after infection by microscopic observation and staining with a 5% crystal violet solution. The viable and death cells ratio was calculated. The MOI at which only 30% of cells were dead was considered suitable.

### 2.7 Adhesion assay

HUF cells were precultured overnight in a BME medium containing 2% FBS at a density of 500,000 cells/well. Bacterial cells were centrifuged at 10,000 rpm/10 min, and the culture medium residue was discarded. The pellets were resuspended in fresh BME w/2% FBS medium, and the HUF cells were infected at the calculated MOI. At 24 hours post-infection, HUF cells were washed with DPBS and stained for 15 minutes with BacLight Green fluorescent dye (Invitrogen, Waltham, USA. MA), prepared according to the manufacturer’s instructions. Cells were then trypsinized and collected by centrifugation (5,000 rpm/5min). The supernatant was discarded, and the pellet was suspended in DPBS. Samples prepared in this manner were placed on a flat-bottomed black culture plate. The measurement was performed at a wavelength of Ex/Em = 480/561 nm using the SpectraMax i3 plate reader (Molecular Devices, San Jose, USA. CA).

### 2.8 ROS level measurement

HUF cells were precultured, as described above. Bacterial cells were centrifuged at 10,000 rpm/10 min, and the culture medium residue was discarded. Microbial cells were suspended in fresh BME w/2% FBS medium. Next, HUF cells were infected with bacterial inoculum at calculated MOI and cocultured for 24 hours. Then, the cell monolayer was washed with DPBS, and H_2_DCF-DA dye (ThermoFisher Scientific, Waltham, USA. MA) in BME medium w/o FBS was added. Incubation with the dye was performed for 40 min at 37°C. After incubation, cells were trypsinized, collected by centrifugation (5,000 rpm/5min), and placed on a flat-bottomed black culture plate. The measurement was performed at Ex/Em = 492/527 nm using the SpectraMax i3 plate reader (Molecular Devices, San Jose, USA. CA)

### 2.9 Statistics

The bioinformatics analysis pathway for data obtained from 16S rRNA amplicon sequencing was described in detail in section 2.4 Bioinformatic sequence analysis, where it was indicated that the analyses were carried out based on the QIIME2 2021.4 platform. The statistics related with *in vitro* examination were performed by using GraphPad Prism 8.

## 3 Results

### 3.1 Participants characteristics

Twenty-nine patients were included in this study. The patients were divided into two major groups: 13 participants with endometrial EM and 16 with EC. The characteristics of cohorts are described in **Table 1**. Material examination and diagnoses were made by a pathologist at the Fryderyk Chopin Clinic no 1 (Rzeszow, Poland). The material from healthy women was not considered in this study because the hysterectomy is not provided in healthy patients. Endometrial myoma was chosen instead because these cells are non-tumorigenic in nature, and at the molecular level, they have qualities closer to normal cells on molecular bases. Significant differences were observed for age (p = 0.002) and thyroid abnormalities (p = 0.043) between the EM and EC groups, with endometrial cancer patients being older and more likely to report thyroid abnormalities.

### 3.2 Statistical comparison of uterus lesions microbiome

**Figure 2:**
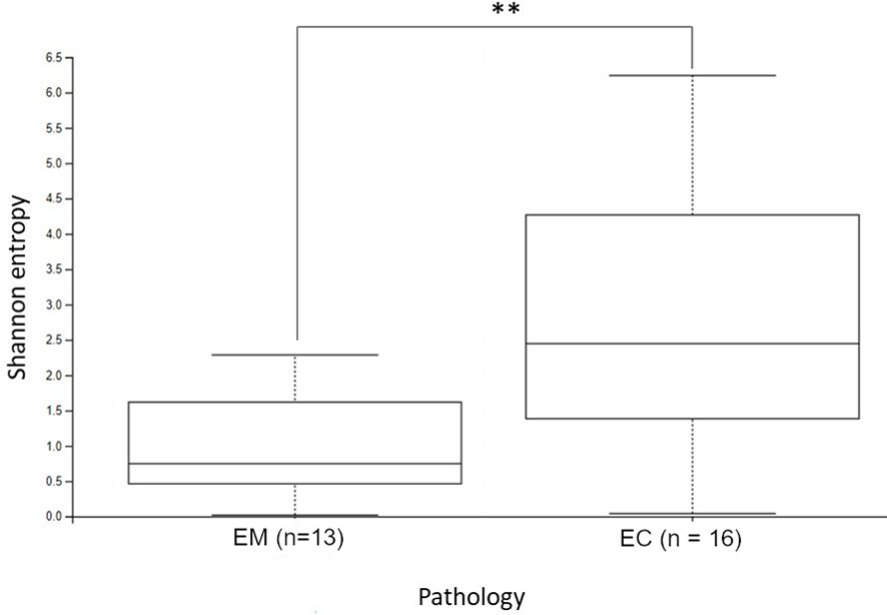
The alpha diversity comparison of EM and EC microbiome, based on the Shannon index. **p < 0.01.

**Figure 3:**
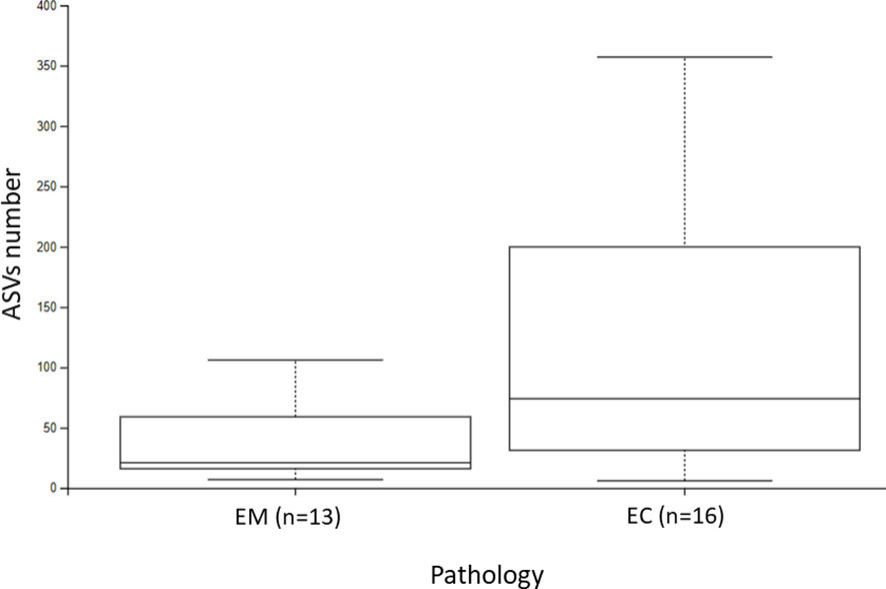
The ASVs number of endometrial myoma (EM) and endometrial cancer (EC) samples.

The α-diversity comparison based on the Shannon index revealed statistically significant differences between EM and EC samples (p = 0.028, p < 0.01) (**Figure 2**). It can be noticed that samples collected from the EC patients were characterized by greater biodiversity than samples from the EM patients, which may suggest the appearance of dysbiosis. The observed amplicon sequence variants (ASVs) number is included in **Figure 3**. It was shown that EM and EC differ statistically significantly in terms of the ASVs number (p = 0.039), wherein a higher number of ASVs is observed in the EC group. In addition to Shannon index and ASVs number, statistical comparison was also performed in an analogous way for Pielou’s evenness and showed a statistically significant difference between the EM and EC groups (p = 0.02).

To provide a comprehensive overview of alpha diversity metrics, a detailed summary of the results, including mean, standard deviation (SD), median, and interquartile range (IQR), is presented in **Table 2**. The table further illustrates the observed differences in Shannon entropy, ASVs number, and Pielou’s evenness between the EM and EC groups. These results indicate that the EC group demonstrates greater richness and variability in the microbiota, consistent across all alpha diversity metrics analyzed.

**Table 2:** Summary of alpha diversity metrics for EM and EC groups, including Shannon entropy, ASVs number, and Pielou’s evenness.

Metric	Group	Mean	SD	Median	IQ	IIIQ	IQR
Shannon entropy	EM	1.17	1.06	0.75	0.46	1.63	1.17
	EC	2.85	1.92	2.45	0.94	4.75	3.81
ASVs number	EM	41.92	35.07	21.00	16.00	77.00	61.00
	EC	121.25	107.51	74.00	27.75	203.25	175.50
Pielou evenness	EM	0.33	0.22	0.28	0.18	0.57	0.38
	EC	0.37	0.22	0.36	0.17	0.53	0.36

EC – samples from patients diagnosed with endometrial cancer; EM – samples from the endometrial microbiota of non-cancer patients; SD – Standard Deviation; IQ – interquartile (The first quartile, Q1, which represents the 25th percentile of the data); IIIQ – interquartile (The third quartile, Q3, which represents the 75th percentile of the data); IQR – interquartile Range (The difference between Q3 and Q1, indicating the spread of the central 50% of the data). Data are presented as mean ± SD, median, and interquartile ranges (IQ, IIIQ, IQR).

The α-diversity results collectively indicate significant differences in the richness, balance, and evenness of the microbiota between the groups, underscoring a potential role of dysbiosis in endometrial cancer pathogenesis.

**Figure 4:**
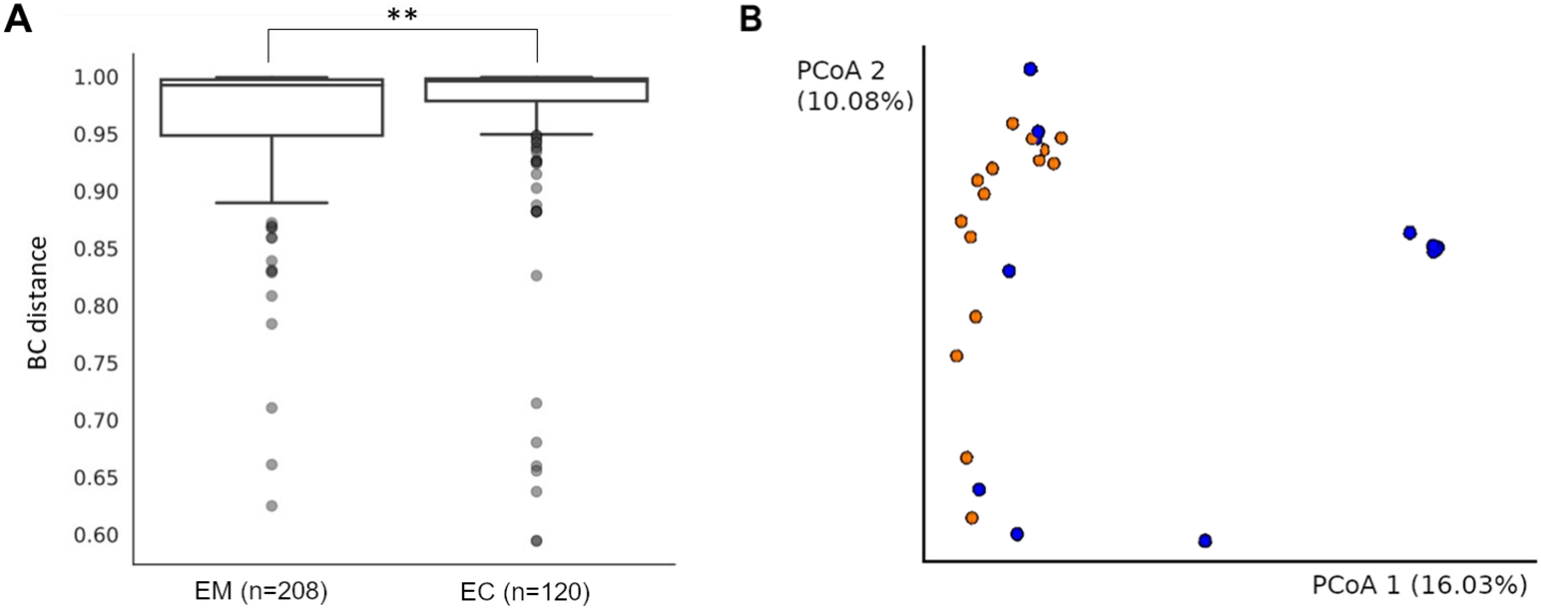
The Bray-Curtis distance comparison of endometrial myoma (EM) and endometrial cancer (EC) samples. ** = p < 0.001 **(A)**. The Bray-Curtis distance mapping of samples. EM – blue; EC – orange **(B)**.

Principal Coordinate Analysis (PCoA), based on Bray-Curtis distance (**Table 3**, **Figure 4A**), showed the significant sample separation according to the pathology type. Moreover, the sequencing results revealed that EM samples were colonized by a homogeneous microbiome dominated by *Lactobacillus* spp. (**Figure 4B**, blue dots). The comparison between the EM and EC groups based on Bray-Curtis distance showed statistically significant differences (p = 0.001).

**Table 3:** The Bray-Curtis distance comparison of endometrial myoma (EM) and endometrial cancer (EC) samples.

Group 1	Group 2	Sample size	Permutations	pseudo-F	*p*-value	*q*-value
EM	EC	29	999	2.647731	0.001	0.0030

**Figure 5:**
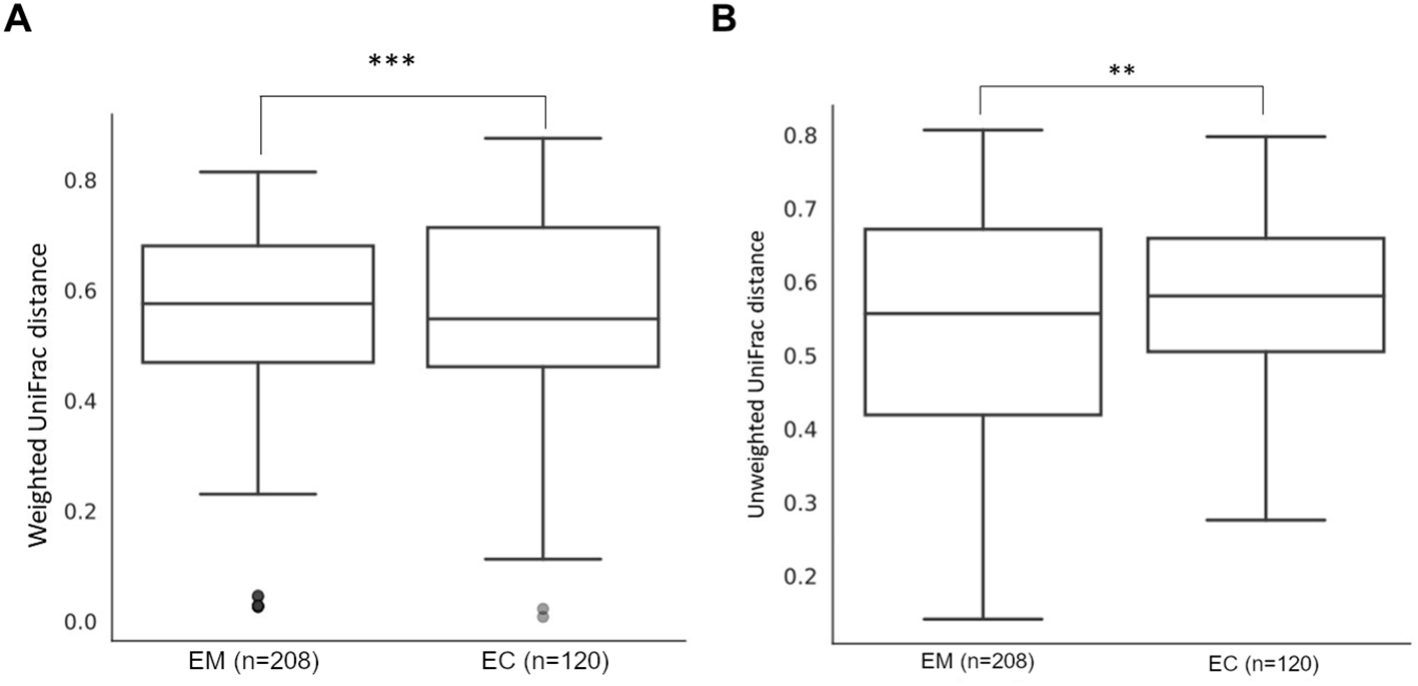
Weighted **(A)** and unweighted **(B)** UniFrac comparison of endometrial myoma (EM) and endometrial cancer (EC) samples. ** = p < 0.01, *** = p < 0.001.

These observations were also confirmed by β-diversity analysis with the use of the Unweighted UniFrac (p = 0.014), as well as with Weighted UniFrac methods (p = 0.005) (**Figure 5**), and the results are presented in **Table 4**.

**Table 4:** Unweighted and weighted UniFrac method comparison of endometrial myoma (EM) and endometrial cancer (EC) samples.

Group 1	Group 2	Sample size	Permutations	pseudo-F	*p-*value	*q*-value
Unweighted UniFrac						
EM	EC	29	999	2.999545	0.014	0.042
Weighted UniFrac						
EM	EC	29	999	4.624288	0.005	0.0150

ANCOM analysis of the samples was performed using the model in which taxa that were identified in less than five samples were removed. The results of taxon analysis were associated with the differentiating effect of d:Bacteria; p:*Firmicutes*; c:*Clostridia*; o:*PeptostreptococcalesTissierellales*; f:*Peptostreptococcales*-*Tissierellales*; g:*Anaerococcus*, which were predominant in the samples from EC. Possibly, the *Anaerococcus* representatives could be the indicators of dysbiosis, and their presence correlates with EC incidence. Detailed data on detection thresholds and prevalence are presented in **Table 5**. Additionally, quantitative analysis revealed significant differences in the average abundance and median presence of *Anaerococcus* between EC and EM groups, as shown in **Table 6**.

**Table 5:** Prevalence of *Anaerococcus* in EC and EM samples at different detection thresholds.

	Present > 0	%	Present > 0.1%	%	Present > 1%	%
**EM**	6	46,154	1	7,692	0	0,000
**EC**	14	87,500	10	62,500	8	50,000

EC – samples from patients diagnosed with endometrial cancer; EM – samples from the endometrial microbiota of non-cancer patients; Present > 0% – detection of *Anaerococcus* in samples at an abundance higher than 0%; Present > 0.1% – detection of *Anaerococcus* in samples at an abundance higher than 0.1%; Present > 1% – detection of *Anaerococcus* in samples at an abundance higher than 1%. The table shows the percentage of samples with *Anaerococcus* detected above thresholds of >0%, >0.1%, and >1% in EC (endometrial cancer) and EM (endometrial microbiota) groups. These data illustrate the significantly higher prevalence of *Anaerococcus* in EC samples.

**Table 6:** Quantitative analysis of *Anaerococcus* abundance in EC and EM samples.

	Mean	SD	Median	IQ	IIIQ	IQR
EM	0,016	0,045	0,000	0,000	0,003	0,003
EC	3,943	5,753	1,184	0,016	5,887	5,872

SD – Standard Deviation; IQ – interquartile (The first quartile, Q1, which represents the 25th percentile of the data); IIIQ – interquartile (The third quartile, Q3, which represents the 75th percentile of the data); IQR – interquartile Range (The difference between Q3 and Q1, indicating the spread of the central 50% of the data). The table presents the mean percentage abundance, standard deviation (SD), median, and interquartile range (IQR) of *Anaerococcus* in EC and EM groups, highlighting the significantly higher abundance in EC samples.

**Figure 6:**
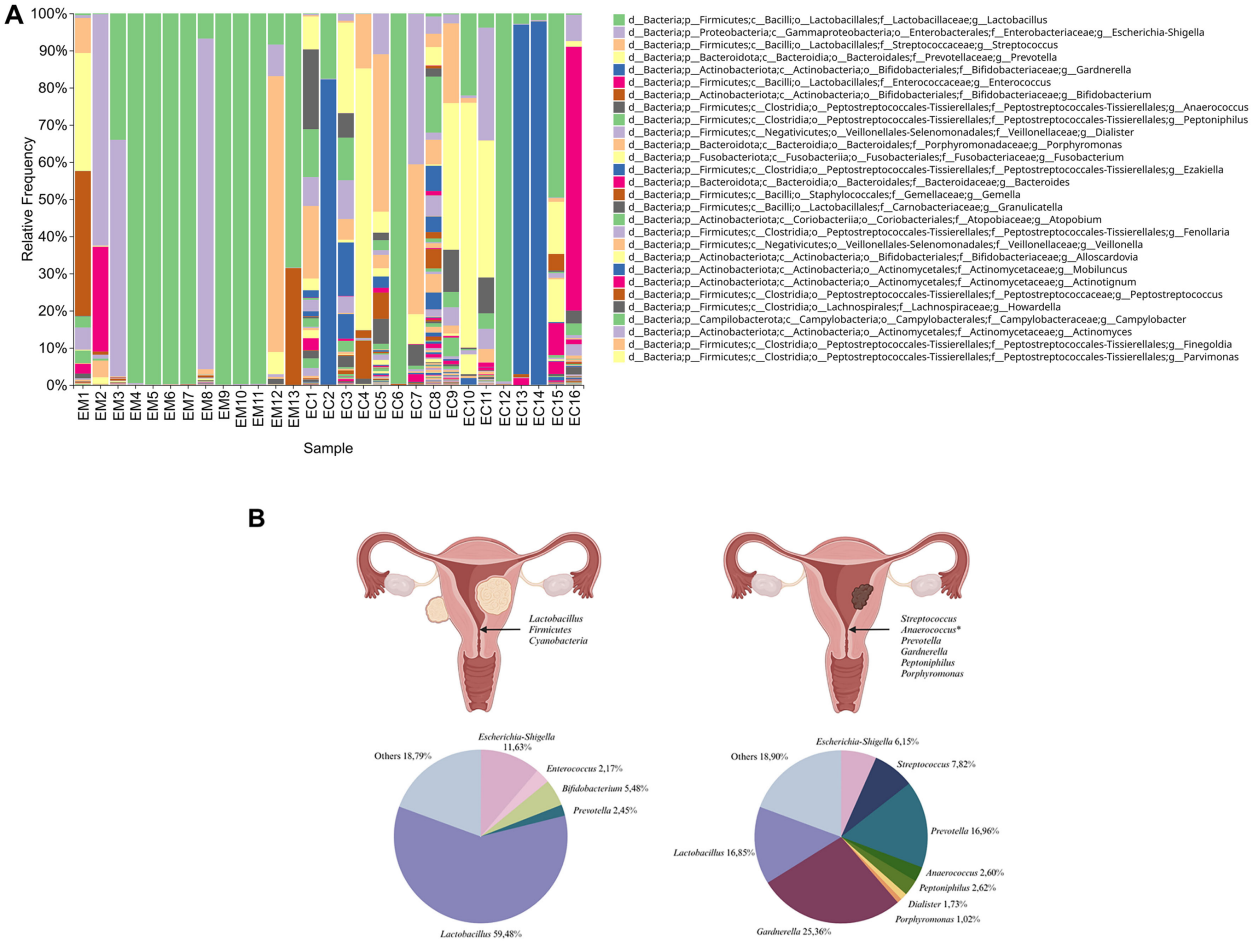
Taxonomic composition of the samples’ microbiota at the genus level. Samples were sorted by groups of endometrial myoma (EM1-EM13) and endometrial cancer (EC1 -EC16) cohorts **(A)**. The main taxons of bacteria identified in the samples from women with endometrial myoma (left) and endometrial cancer (right) and their corresponding percentage distribution in the form of a pie chart **(B)**.

In this analytical model, the sample types differed significantly in the abundance of *Firmicutes* and *Cyanobacteria* in EM. Moreover, the analysis showed the predomination of pathogenic taxons such as *Streptococcus*, *Anaerococcus*, *Prevotella*, *Gardnerella*, *Peptoniphilus*, and *Porphyromonas* to predominate in EC samples (**Figure 6A**). The main types of bacteria in the pathologies studied and their percentage distribution in the form of pie chart are presented in **Figure 6B**.

### 3.3 The adhesion level of *Anaerococcus vaginali*s to human uterus fibroblasts

Based on the above taxonomic analysis of the uterine microbiota, revealing genus *Anaerococcus* as a differentiating taxon between EC and EM, the strain *A. vaginalis* DSM 7457 was selected for the next experimental steps. This strain was originally isolated from an ovarian abscess (Ezaki et al., 2001). Adhesion assays were conducted to assess *A. vaginalis* ability to bind to endometrial cells. Cells were cultured and incubated with *A. vaginalis* for 24-hours, and non-adherent bacteria were washed off. Adhesion was quantified via BacLight Green dye fluorescence measurement. Appropriate control, such as physiological vaginal flora representative *L. jensenii*, was included to validate the results.

**Figure 7:**
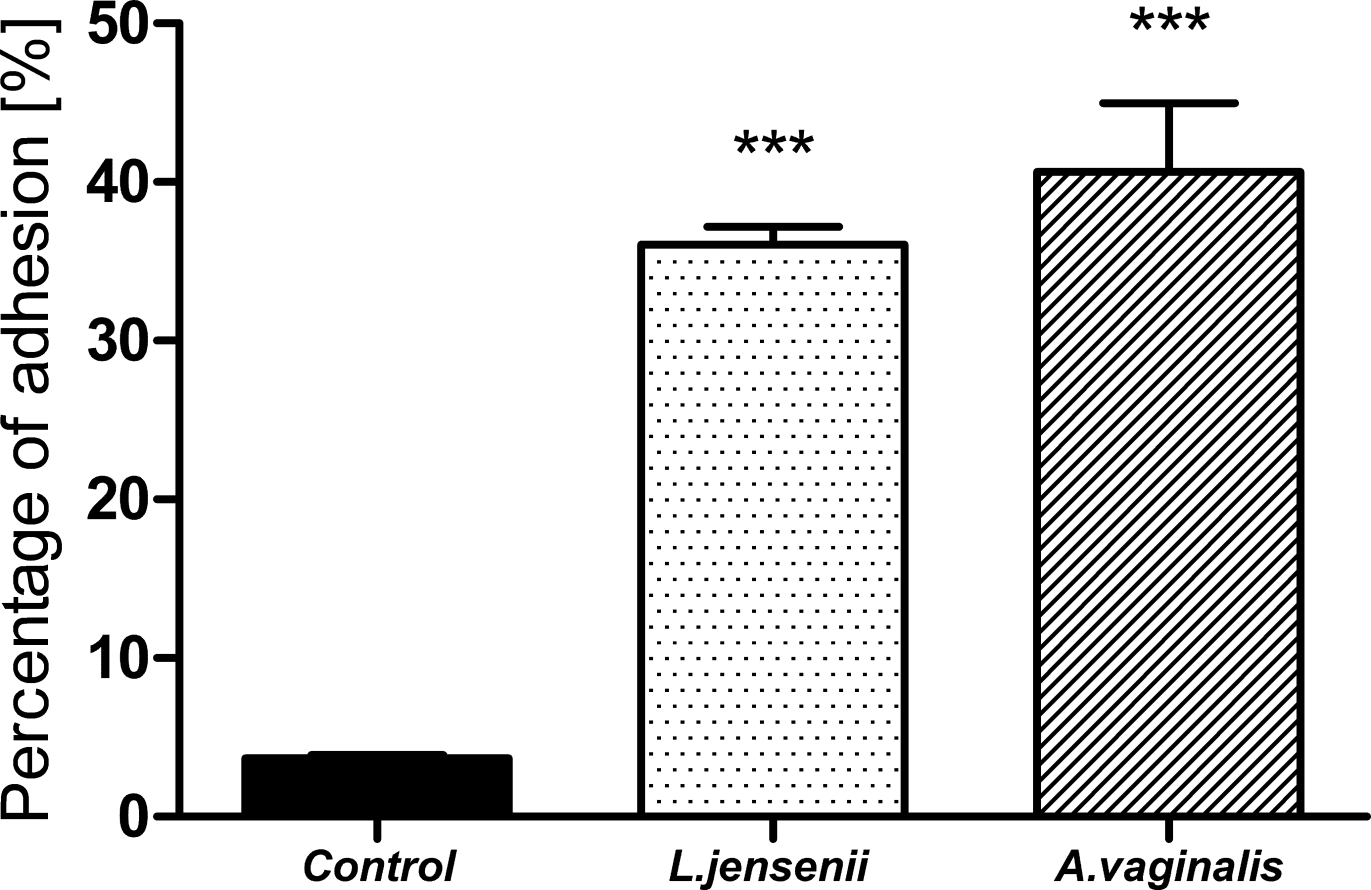
Percentage of *L. jensenii* and *A. vaginalis* adhesion to HUF cells at 0.5 MOI of *L. jensenii* and *A. vaginalis*. Data were obtained by measuring fluorescence at Ex/Em = 480/561 nm in three independent experiments. Results were statistically compared with the control using One-way ANOVA with Bonferroni multiple comparison test. ***p < 0.001.

The results of the test showed a high adhesion rate for both *L. jensenii* and *A. vaginalis* strains (**Figure 7**). However, the tendency of a higher adhesion rate of *A. vaginalis* to HUF cells compared to *L. jensenii* was observed. The percentage of adhesion of the studied pathogenic strain was almost 50% after 24 hours of infection. The MOI of 0.5 was found to be suitable for testing, as the result of calculations described in methodology.

### 3.4 The ROS level changes in HUF cells after *Anaerococcus vaginalis* infection

**Figure 8:**
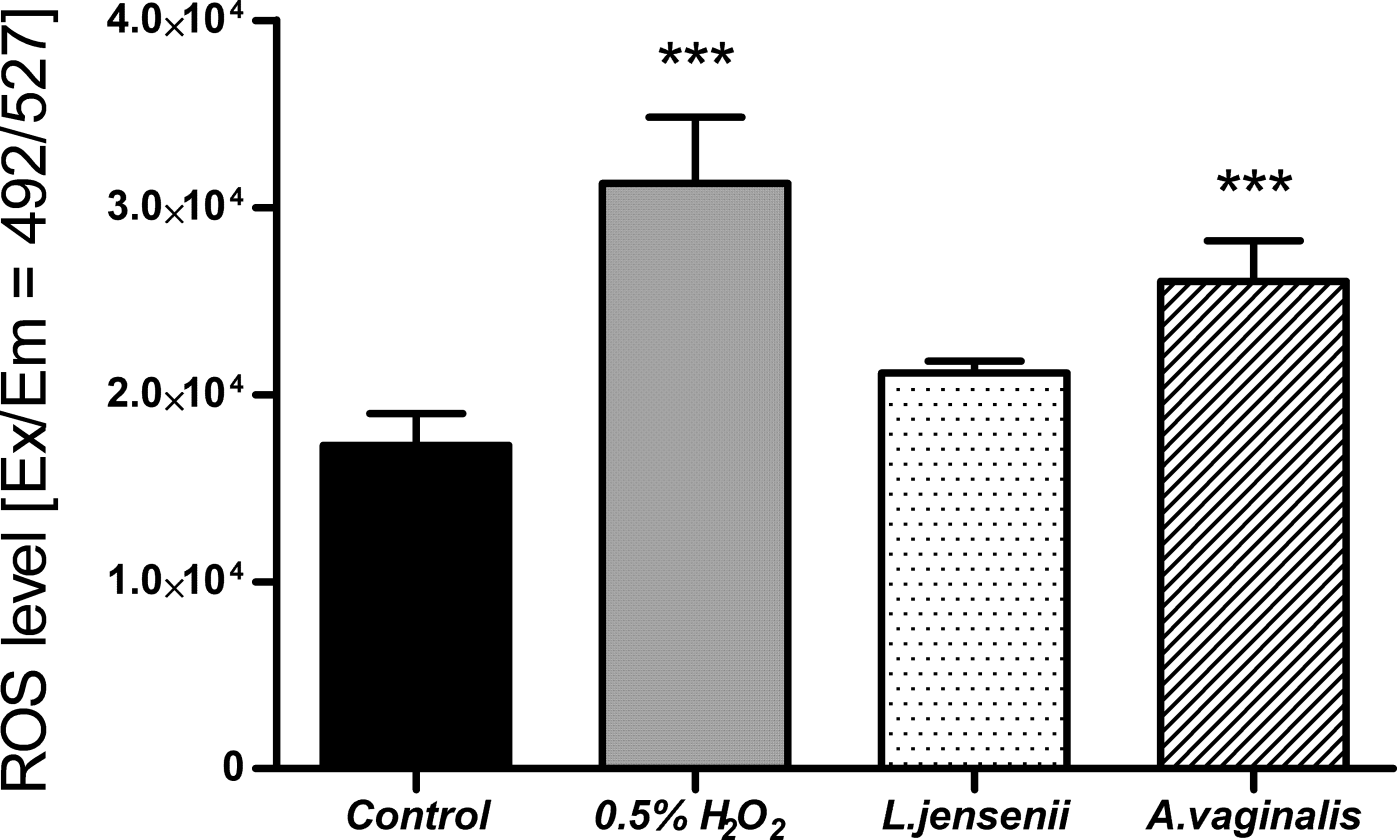
ROS level in HUF cells, after 24-hour co-incubation at 0.5 MOI of *L. jensenii* and *A. vaginalis*. Data were obtained by measuring fluorescence after H2DCFDA staining at Ex/Em = 492/527 nm in three independent experiments. Results were statistically compared with the control using a One-way ANOVA with Bonferroni multiple comparison test; *** = p < 0.001; *L. jensenii* was used as a representative of the physiological state, and the 0,5% H_2_O_2_ as the positive control (ROS inducer).

The increase in reactive oxygen species (ROS) level is one of the indicators of the inflammatory process that could be associated with infections caused by pathogens. It is one of the responses of the host’s innate immunity to microbial invaders and is aimed at eliminating pathogens. However, long-term exposure to bacterial infection could lead to repercussions - the accumulation of damage in the human cellular apparatus. These abnormalities could lead to the development of cancer (Kennel and Greten, 2021; Spooner and Yilmaz, 2011). We performed ROS production assays using non-malignant human endometrial cells (HUF) exposed to *A. vaginalis*. ROS levels were quantified using DCFH-DA fluorescence measurement, following describe previously protocol. Controls included untreated cells and cells exposed to L. jenseni, as well as 0,5% H2O2. The level of ROS in HUF cells 24 h after infection with *A. vaginalis* are shown in **Figure 8**.

The results of ROS level measurement in HUF cells after 24 hours of infection with *A. vaginalis* show a statistically significant increase. This level was statistically similar to the one induced by 0.5% H_2_O_2_. Such effect was not observed when HUF cells were infected with the *L. jensenii*, indicating a reduced impact on oxidative stress induction in human cells. The observed increase in ROS levels in uterine fibroblasts in response to *Anaerococcus vaginalis* infection indicates early inflammatory changes. However, this study is limited to an acute infection model. Future research should incorporate inflammatory markers analysis such as IL-6 and TNF-α, which could provide valuable insights into the role of inflammation in EC progression.

## 4 Discussion

Since the progress of sequencing techniques, microbiome studies found practical applications in cancer research. It has been shown that microbial pathogens have a tumorigenic effect in 15-20% of reported cancer cases and are referred to as “oncomicrobes.” The risk of pathogen influence on carcinogenesis and progression is also present in the so-called “complicit” microbes, whose functions are broad and yet not well understood (Menati Rashno et al., 2021; Sepich-Poore et al., 2021). The pathogenic changes of microbiota can promote resistance to host cell death, which is one of the cancer hallmarks, and induce cancer-promoting inflammation. The composition of the bacterial microbiome modulates the specific alternative responses, one of which depends on the inflammasome complex and the second on the inflammasome independent secretion of pro-inflammatory cytokines, highlighting the key role in activating different degrees of inflammation (De Seta et al., 2019). In addition, the microbiota influences carcinogenesis by releasing carcinogenic molecules (e.g., genotoxins) and producing tumor-promoting metabolites. It is undeniable that the microbiome has serious implications for human health and the progression of disease (Menati Rashno et al., 2021; Schwabe and Jobin, 2013).

Endometrial cancer is one of the most common cancers among women in high-income countries. There are many known risk factors: the excessive, uncontrolled exposure of the endometrium to estrogens, including uncontrolled estrogen therapy, early menarche, late menopause, tamoxifen therapy, nulliparity, infertility or failure to ovulate, polycystic ovary syndrome, diet, and general lifestyle. Other risk factors include increasing age, obesity, hypertension, diabetes mellitus, and hereditary nonpolyposis colon cancer. It was found that menopausal age was positively associated with EC, and the risk increased for women of age above 46.5 years (Braun et al., 2016; Wu et al., 2019). Until the second half of the 20th century, the uterine cavity was considered free of microbes. Then it was assumed that colonization of the uterine cavity occurs from the intestine, oral cavity, bloodstream and vaginal mucosa. However, there is increasing evidence of the unique microbial composition of the female reproductive tract (Benner et al., 2018; Toson et al., 2022).

The upper genital tract of healthy women is dominated by *Lactobacillus* species. Studies by De Seta et al. showed the relationship between the presence of lactobacilli and the level of immunological mediators in the vagina (De Seta et al., 2019). This scheme could also be possible in the upper parts of the genital tract. The samples were grouped according to community state types (CSTI-IV), where CSTI was characterized by the presence of *L. crispatus*, CSTII - *L. gasseri*, CSTIII - *L. iners*, and CSTIV by a low number of lactobacilli. Several cross-sectional studies have revealed the interplay between lactobacilli dysbiosis and the changes in microbial composition in the EC (Lu et al., 2021; Walsh et al., 2019; Walther-António et al., 2016).

In our study, we identified the endocervical canal microbiomes of the 29 participants with EC or EM. The inclusion of additional alpha diversity metrics (**Table 2**), such as ASVs number and Pielou’s evenness, allowed for a more nuanced understanding of microbial diversity and distribution in EC and EM samples. The significantly higher ASVs number observed in EC samples aligns with the hypothesis of a more diverse and potentially pathogenic microbiota in cancerous environments. The endometrial communities of women in both groups markedly differed from one another in terms of bacterial species composition. We observed that EM samples had homogeneous microbiomes dominated by *Lactobacillus*, while the EC group featured a higher diversity of microorganisms. Our results are consistent with the findings of Mitchell et al (Mitchell et al., 2015), who reported that the most commonly detected taxon in the group of 58 women undergoing hysterectomy for noncancer indications was the genus *Lactobacillus*. Moreover, the recent study conducted by Kaakous et al (Kaakoush et al., 2022). indicated that EC was associated with decreased abundance of *Lactobacillus* genus in the group of 70 postmenopausal women undergoing hysterectomy due to the benign pathology or EC. So far, several studies have indicated that disruption of microbial homeostasis may promote many gynecological malignancies and inflammation, eventually leading to carcinogenesis. When the microbial load is too rich in representatives of certain taxa, excessive tissue destruction or immune stimulation can occur, being the stimulus of gynecological diseases. Indeed, our study confirmed that the samples taken from patients with EC were enriched in certain pathogenic taxa such as *Streptococcus*, *Anaerococcus*, *Prevotella*, *Gardnerella*, *Peptoniphilus* and *Porphyromonas*, which is also consistent with the existent literature of endometrial microbiota composition among patients with EC. In a recent study conducted on a group of 28 postmenopausal women undergoing hysterectomy, Wang et al (Wang et al., 2022). proved that although *Lactobacillus* and *Gardnerella* were the dominant bacterial taxa in both EC and adjacent non-EC tissue samples, only the EC samples were enriched in such genera as *Prevotella*, *Atopobium*, *Anaerococcus*, *Dialister*, *Porphyromonas* and *Peptoniphilus*.

Nowadays, more and more studies point to the presence of specific strains as potential indicators of EC. Microorganisms in the stage of dysbiosis, especially some certain pathogenic taxa, may stimulate the immunopathological processes and promote destabilization of the host genome by releasing bacterial secondary metabolites, which can damage human genetic material. In a recent study, Li et al (Li et al., 2021). confirmed that the increasing abundance of *Prevotella* in endometrial tissue, especially when correlated with the elevated level of serum D-dimer (DD) and fibrin degradation products (FDPs), may be an important factor associated with carcinogenesis. A study conducted by Lu et al (Lu et al., 2021). indicated a correlation between an increased abundance of *Micrococcus* and IL-6 and IL-17 mRNA levels in EC patients, which suggests the pro-inflammatory role of these microorganisms in tumor genesis. In another study, Walter-Antonio et al (Walther-António et al., 2016). revealed that the simultaneous presence of *Atopobium vaginae* and *Porphyromonas somerae*, especially if combined with an increased vaginal pH (>4,5), was associated with EC in a group of 31 patients with EC, endometrial hyperplasia or benign uterine conditions. In a study of 148 women undergoing hysterectomy due to benign disease, endometrial hyperplasia or EC, Walsh et al (Walsh et al., 2019). also identified *P. somerae* as the most predictive microbial marker of EC. Moreover, Caselli et al (Caselli et al., 2019). revealed that *P. somerae* and *A. vaginae* induced the release of proinflammatory cytokines by human endometrial cells after 24 hours of co-culturing. As indicated by the above studies, enrichment in certain taxa was observed among patients suffering from hyperplasia and cancer, suggesting an inflammatory role of specific microorganisms in carcinogenesis.

The presence of certain bacterial species may contribute to altering the expression of genes encoding proteins involved in the inflammatory response, proliferation or apoptosis. Consequently, these events may ultimately disrupt physiological processes and promote the development of disease states, such as endometritis and endometriosis, which are associated with the development of cancer. In the study described herein, using ANCOM analysis of the 16S rRNA metagenomics data, we identified *Anaerococcus* as the taxon differentiating between EC and EM microbiomes. The increased prevalence and abundance of *Anaerococcus* in EC samples, as demonstrated in **Tables 5** and **6**, supports its potential involvement in the pathogenesis of endometrial cancer. While correlation does not imply causation, these data strengthen its candidacy as a biomarker and underline the need for further functional studies. In the study conducted by Tsementzi et al (Tsementzi et al., 2020), samples of vaginal swabs from women suffering from gynecological cancer who were undergoing pre-radiation therapy (N=65), post-radiation therapy (N=25) and from a control group of healthy volunteers (N=67) were sequenced. Results from 16S rRNA V4 region sequencing revealed that the swabs taken from the cancer group were enriched in 16 phylogroups associated with BV and inflammation, including *Sneathia*, *Prevotella*, *Peptoniphilus*, *Fusobacterium*, *Anaerococcus*, *Dialister*, *Moryella* and *Peptostreptococcus*, comparing to the healthy group. In another study, Liu et al (Liu et al., 2019). collected and sequenced endometrial biopsy tissue and fluid from 130 infertile women with chronic endometritis, revealing that this malignancy is associated with the abundance of bacterial taxa including *Dialister*, *Bifidobacterium*, *Prevotella*, *Gardnerella* and *Anaerococcus*. Another study conducted by Perrotta et al (Perrotta et al., 2020). revealed that in a group of 59 patients with different types of endometriosis, the amount of *Lactobacillus* species was decreased while the vaginal pathogenic bacteria, including *Anaerococcus*, was increased, compared to 24 control samples from healthy volunteers’ group. Moreover, in a recent study, Semertzidou et al (Semertzidou et al., 2024). compared the microbiota composition from 24 benign diseases and 37 EC patients from different anatomical sites (vagina, cervix, endometrium) and observed a high diversity and *Lactobacillus* depletion in the EC group. EC samples were also enriched in several pathogenic species, including *Porphyromonas*, *Prevotella*, *Peptoniphilus*, and *Anaerococcus*.

Based on our sequencing data, we have selected *A. vaginalis* as a representative of the taxon differentiating between EC and EM. While previous studies have identified *Prevotella* and *Gardnerella* as components of dysbiotic uterine microbiomes in EC, these microorganisms are also frequently associated with bacterial vaginosis, a condition prevalent among non-cancerous cohorts. Their presence may therefore reflect generalized microbial dysbiosis rather than EC-specific pathogenesis. In contrast, our study is the first to explore *Anaerococcus* as a first-placed potential contributor to endometrial carcinogenesis. By demonstrating its ability to adhere to endometrial cells and elevate ROS levels *in vitro*, *A. vaginalis* emerges as a unique candidate for further investigation. *Lactobacillus jensenii* was used as a member of the genus *Lactobacillus*, found in the physiological microflora of the uterus. Both strains showed a high adhesion rate, which means they can efficiently colonize endometrium, and the imbalance between lactobacilli and pathogenic species such as *A. vaginalis* may lead to dysbiosis. There is a significant gap in *in vitro* studies investigating the direct effects of *Anaerococcus* on human endometrial cells. Current knowledge is limited to initial observations, such as ROS production and bacterial adhesion, as demonstrated in this study. These findings underscore the need for comprehensive investigations into the molecular mechanisms underlying *A. vaginalis* interactions with host cells, particularly in the context of inflammation or dysbiosis.

Bacterial infections can promote genetic instability of nearby host cells through bacterial genotoxins or tumor promoting metabolite secretion, and the relationship between chronic infections and cancer has already been demonstrated, e.g. in the case of *Helicobacter pylori* and *Fusobacterium nucleatum* in gastric and colorectal cancers, respectively (Kostic et al., 2012; Lofgren et al., 2011; McCoy et al., 2013; Touati et al., 2003). Moreover, bacterial infections usually lead to inflammation, causing ROS induction, dysregulation of the innate immune system and cell damage. However, the association between inflammation and cancer is not fully understood (Coussens and Werb, 2002; Hussain et al., 2003; Lin and Karin, 2007). Dossus et al. conducted a case-control study which comprised 305 incident cases of EC and 574 matched controls, where they observed a significant increase in the risk of EC with elevated levels of three inflammatory markers: C-reactive protein (CRP), interleukin 6 (IL6), and IL1 receptor antagonist (IL1Ra) (Dossus et al., 2010). In addition to inflammatory markers, free radicals are generated during the inflammation response to eliminate invading photogenes (Spooner and Yilmaz, 2011). However, elevated levels of ROS can also damage healthy human cells and trigger carcinogenesis (Hussain et al., 2003). It has been estimated that infections and associated inflammation contribute to about 15% of all cancer cases worldwide (Coussens and Werb, 2002). In our research, we have demonstrated that *A. vaginalis* induced a significant increase of ROS inside human uterine fibroblasts, and the levels were much higher than in the presence of *L. jensenii.* High levels of ROS may damage proteins, lipids, membranes, and organelles. The oncogenic role of ROS is associated with the induction of oxidative DNA damage involving double-stranded breaks and the formation of 8-oxo-7,8-dihydro-2′-deoxyguanosine (8-oxodG). This oxidized guanine induces transversion of guanine to thymine and is a major cause of spontaneous mutagenesis, leading to carcinogenesis (Nakamura and Takada, 2021; Valavanidis et al., 2009). Moreover, ROS as the one of the inflammation inducers, could be a key contributor to carcinogenesis (Coussens and Werb, 2002). Inflammatory markers such as interleukins (e.g., IL-6, IL-8), TNF-α, and other chemokines could also provide additional evidence of the inflammatory environment in EC patients. Future studies incorporating measurements of these markers in serum or conditioned media from *in vitro* models could help establish a more comprehensive understanding of the interplay between inflammation, ROS levels, and microbial dysbiosis.

The literature data and the results of 16S rRNA metagenomics presented in this study indicated significant differences in the composition of the uterine microbiome between cases of EC and benign lesions of this organ. Our results are not only in agreement with data indicating the pathological involvement of the genus *Anaerococcus* in the development of the most common gynecological diseases but also shed new light on our previous understanding regarding the possibility of induction of neoplastic processes by this microorganism. This knowledge may lead to new diagnostic and preventive strategies for EC, but clarifying the role of *A. vaginalis* in EC requires further research. If confirmed in the larger cohorts, examination of the microbiome of cervical swabs concerning the presence of *A. vaginalis* as an EC indicator could form the basis of microbiological diagnosis of EC, which would be of great importance in detecting the early stages of the disease.

## 5 Conclusions

In conclusion, our study indicated that microbial composition significantly differs between patients with endometrial cancer and benign lesions such as myoma. Furthermore, some pathogenic taxa, such as *Streptococcus*, *Anaerococcus*, *Prevotella*, *Gardnerella*, *Peptoniphilus*, and *Porphyromonas*, may play a key role in endometrial cancer development and progression. Understanding the functional interactions between the endometrial microbiome and the immune system could provide new insights into the pathogenesis of endometrial cancer. Despite the limitations, such as the limited sample size, our study provides two key contributions to the field. First, it introduces *Anaerococcus* as a novel genus associated with EC, offering a new avenue for biomarker research. The presence of specific microbial signatures, including *Anaerococcus*, could serve as early indicators of endometrial cancer, aiding in timely diagnosis. Second, by evaluating *A. vaginalis* potential to drive ROS production and adhere to non-malignant endometrial cells, we establish a foundation for understanding its functional role in EC pathogenesis. These findings distinguish *A. vaginalis* from previously studied genera and emphasize its diagnostic potential. Understanding the role of *Anaerococcus* in EC may lead to novel therapeutic interventions aimed at modifying the uterine microbiome to prevent or treat cancer. While further research is required to validate these findings, this study marks an important step toward identifying microbial signatures that may enhance the early diagnosis of EC.

## Data Availability

The data presented in the study are deposited in the NCBI Sequence Read Archive (BioProjects), accession number PRJNA1203774. The repository can be accessed at the following link: https://www.ncbi.nlm.nih.gov/bioproject/?term=PRJNA1203774. Additionally the key raw data has been validated onto The University of Lodz Repository: http://hdl.handle.net/11089/52937.
